# Remember the time

**DOI:** 10.1111/ppl.13823

**Published:** 2022-12-04

**Authors:** Sam W. van Es

**Affiliations:** ^1^ Department of Plant Physiology Umeå University Umeå Sweden

## Abstract

Like the ability of a fox to avoid the same snare twice, the plant *Arabidopsis thaliana* seems to be able to respond differently to an event it has previously encountered. Shrewdness is no surprising characteristic of a fox, but the comparison does invite the question: do plants possess memory? In this edition of Physiologia Plantarum, the article by Vyse et al. (2022) describes how Arabidopsis responds differently to cold‐stress, when encountering it for the first time (priming) and to a second similar stress after a memory phase (triggering). Arabidopsis seems to perceive the stress after triggering as a milder one and the authors postulate that the memory of the first occurrence of the stress reduces the amount of resources needed to cope with the cold‐stress a second time.

An obvious lack of a (plant)brain makes the idea of a memory in plants difficult to fathom, however, there is quite some experimental evidence for it. In the years preceding his dissertation, Harvey (1918) was one of the first to scientifically approach hardening, a practice used by plant growers for centuries to prepare vulnerable seedlings for low temperatures. Hardening ensures that every time the seedling gets placed back in a protected environment after a day in harsher outdoor conditions, it gets a little better prepared for the latter. Without some form of memory, the plant would experience each day outdoors as its first. Another well‐known example of memory is the heat shock response, it was first discovered in Drosophila in the 1960s, and has subsequently been described in a wide range of organisms including plants (see review by Vierling [1991]). This mechanism allows a plant to deal with a lethal heat‐stress, provided that it has been primed with a nonlethal stress first. The plants memorize the initial nonlethal stress, allowing the plant to survive exposure to an otherwise lethal heat‐stress several days later.

These are examples of plant memory that lasts a few days, at most, and are largely attributed to changes in gene expression and protein accumulation. There are also reports of plants passing a form of epigenetic memory of a stressful condition down to their offspring. One such example is mutations in the plant specific MSH1 protein, possibly leading to changes in DNA methylation, that led to different coping strategies such as growth adjustment and changes in flowering time, which could be passed down several generations (Yang et al., 2020).

Having a memory of a stress event is beneficial to a plant, since the initial response to a stress (priming) is rather taxing; it uses up valuable resources and reduces the survival rate and reproductive success, and generally severely reduces plant growth and development. That's why the memory phase and response to a repeated stress (triggering) are important, plants can fine tune their response without having to spend the resources they would when the stress was encountered for the first time: minimizing the impact of the stress on their growth and development.

In this issue of Physiologia Plantarum, Vyse et al. (2022) investigate the different response of a plant to cold stress, comparing the initial response (priming) to the response after a memory phase (triggering). The authors conclude that the initial priming response consists of the well‐known CBF regulon (Park et al., 2015): upregulation of the transcription factors CBF1, CBF2, and CBF3 that induce a cascade of cold‐regulated genes. The response to triggering seems to involve CBF4, DDF1, DDF2, and CBF2. These transcription factors are quickly upregulated upon triggering, followed by genes responsible for ROS detoxification, cell wall remodeling, and the production of antifreeze proteins (Figure 1). The authors hypothesize that the different response after triggering allows the plant to fine‐tune their response to cold, without having to spend all the resources and energy they would when initiating the CBF‐regulon.

**FIGURE 1:**
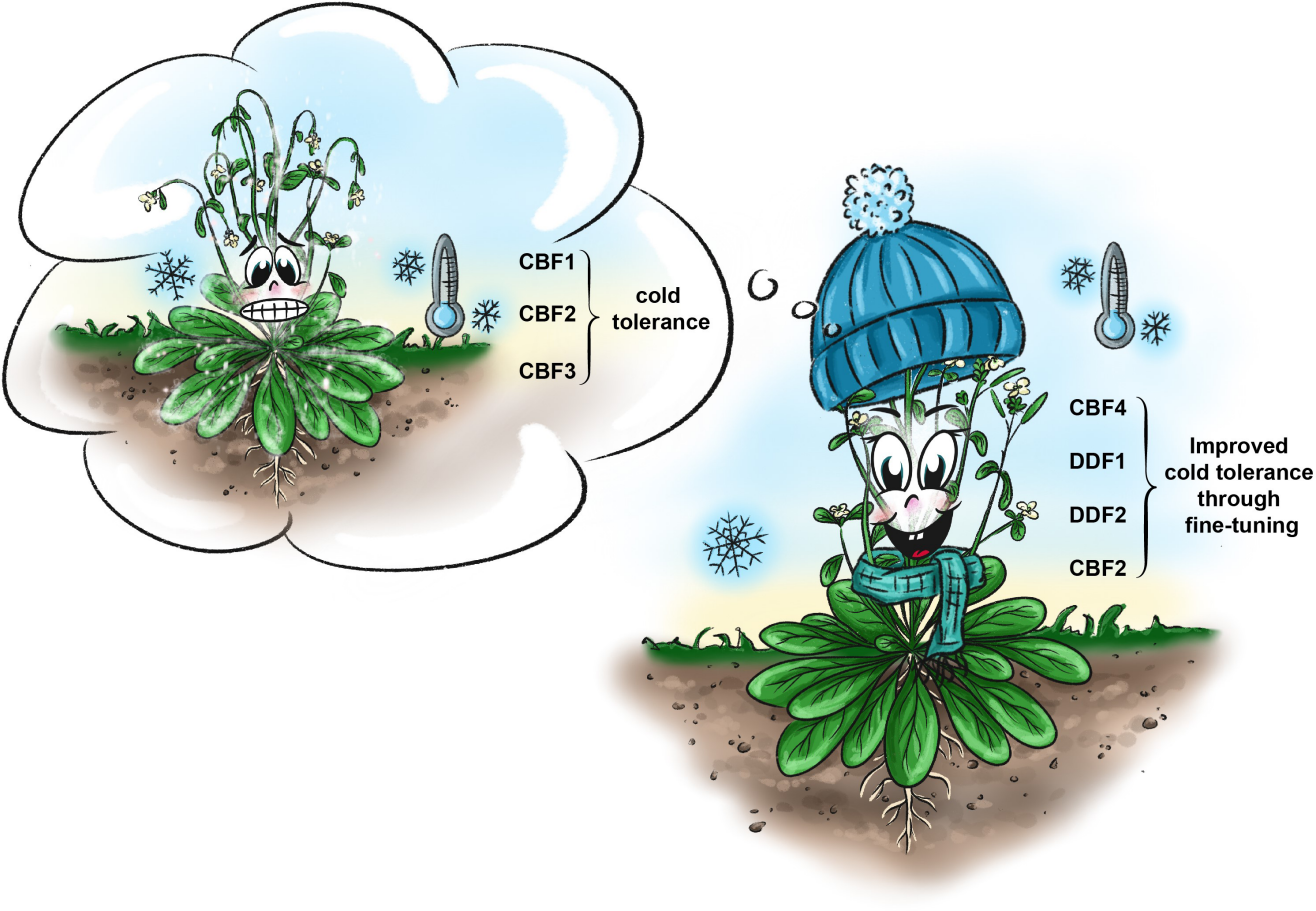
The different response of *Arabidopsis thaliana* to cold‐stress: Priming versus triggering. The initial priming response consists of the upregulation of the transcription factors CBF1, CBF2, and CBF3 that induce a cascade of cold‐regulated genes, leading to the initial cold tolerance, shown here as the “memory.” The response to triggering seems to involve the upregulation of transcription factors CBF4, DDF1, DDF2, and CBF2, which then improve cold tolerance through fine‐tuning several processes. Illustration done by Daria Chrobok, DC SciArt

Research into plant cold responses and frost tolerance is becoming increasingly important since, counterintuitive as it may sound, the changing and warming climate increases the chance of spring frost‐damage in plants. As spring starts earlier, plants will expose their young leaves to the elements earlier in the year, increasing the risk of damage by a late‐spring frost. It is estimated that as much as 35% of the European temperate forests will suffer increased late‐spring frost damage, and in North America and Europe, late‐frost damage to crops and trees causes more economic losses to agriculture than any other climate‐related hazards (Zohner et al., 2020).

Knowing how plants respond to cold, and how a memory changes their response, might translate to breeding programs for cold‐resistant plants. The research presented in the paper by Vyse et al. (2022) has contributed to this.
